# Non ST-segment elevation myocardial infarction in patient with essential thrombocythemia

**DOI:** 10.1186/1477-9560-7-1

**Published:** 2009-02-20

**Authors:** Emin Alioglu, Nurullah Tuzun, Fahri Sahin, Buket Kosova, Serkan Saygi, Istemihan Tengiz, Ugur Turk, Nazan Ozsan, Ertugrul Ercan

**Affiliations:** 1 Central Hospital, Department of Cardiology, Izmir, Turkey; 2 Ege University School of Medicine, Department of Hematology, Izmir, Turkey; 3 Ege University School of Medicine, Department of Medical Biology, Izmir, Turkey; 4 Karsiyaka State Hospital, Department of Cardiology, Izmir, Turkey; 5 Ege University School of Medicine, Department of Pathology, Izmire, Turkey; 6 Canakkale Onsekiz Mart University, Faculty of Medicine, Department of Cardiology, Canakkale, Turkey

## Abstract

A 68-year-old woman presented with acute chest pain and a greatly increased platelet count. Cardiac catheterization revealed subtotal occlusion and a thrombus-like filling defect in the right coronary artery. The patient was successfully treated with intravenous tirofiban. Essential thrombocythemia was diagnosed based on bone marrow findings, clinical presentation and laboratory analysis. The relationship between intracoronary thrombus and essential thrombocythemia is discussed.

## Introduction

Essential thrombocythemia (ET) is a clonal disorder of unknown etiology involving a multipotent hematopoietic progenitor cell and is manifested clinically by the overproduction of platelets in the absence of a definable cause [1]. In 2008, the World Health Organization accepted the presence of the JAK2V617F mutation as a new diagnostic criterion for myeloproliferative neoplasms (MPNs) like polycythemia vera (PV), essential thrombocythemia (ET) and primary myelofibrosis (PMF) [2]. Platelets in this disorder also tend to be dysfunctional, in addition to being elevated in number [3]. The disease is associated with an elevated risk of thrombosis, hemorrhage, and vasomotor symptoms. Thrombotic events are not common but are usually deep venous thrombosis and pulmonary emboli [4,5]. Coronary artery involvement is uncommon. We present a case of coronary thrombus involving the right coronary artery in a patient with ET.

## Case presentation

**Figure 1:**
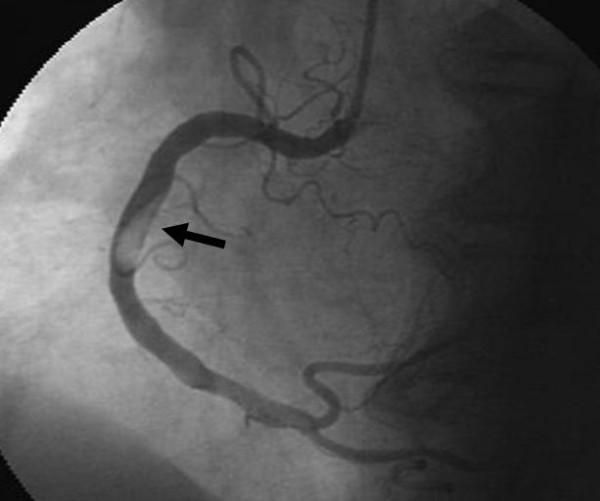
**Coronary angiography revealing thrombus-like filling defect in the mid portion of RCA**.

**Figure 2:**
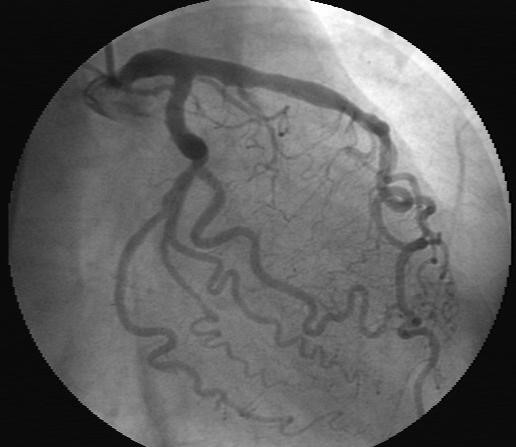
**Left coronary angiography revealed no significant stenosis**.

**Figure 3:**
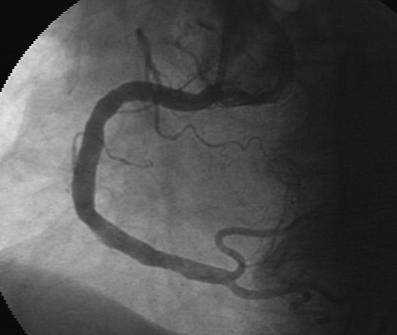
**Coronary angiography revealing RCA free of thrombus**.

**Figure 4:**
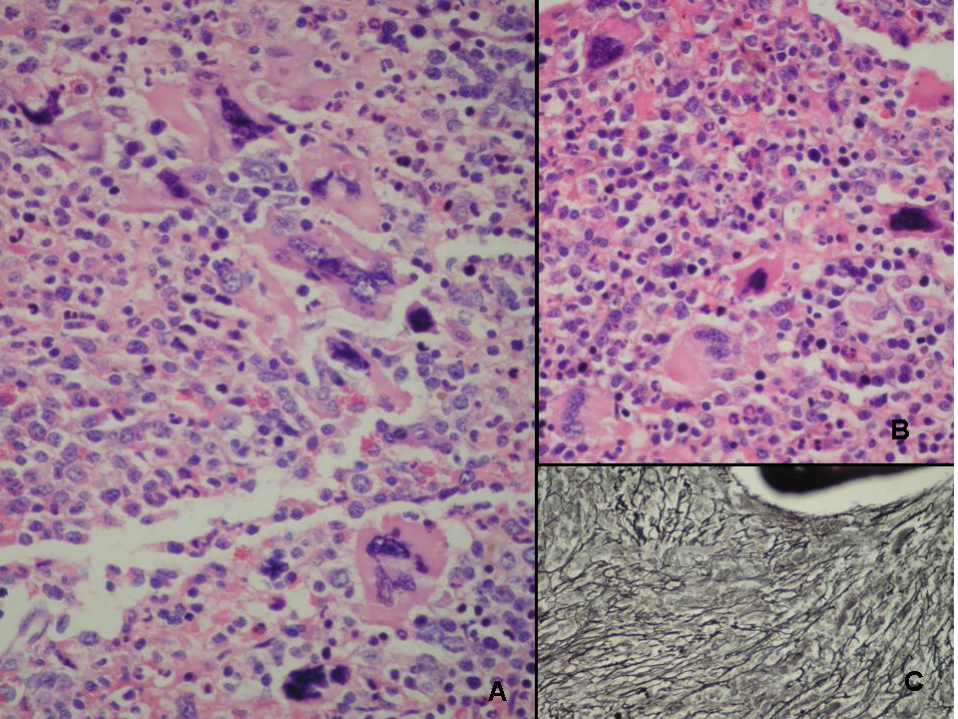
**Bone marrow aspirate showed myeloid and megakaryocytic and megakaryocytic hyperplasia (A&B) (Hematoxylin&Eosin40) with mild degree fibrosisi (Reiculin stain×20)**.

A 68-year-old woman was admitted to our institution because of severe and sudden-onset chest pain. She underwent coronary angiography a year before which revealed a normal right coronary artery (RCA) with nonsignificant atherosclerotic lesions involving the other remaining coronary arteries. Her past medical history was significant for high platelet counts with a nonrevealing workup for myeloproliferative disease, and diabetes which was controlled medically. The patient's blood pressure on presentation was 135/70 mmHg with a heart rate of 70 beats/minute. Physical examination revealed splenomegaly without any other significant pathological findings. Electrocardiography showed regular sinus rhythm with ST segment depression in the inferior derivations. The patient was diagnosed as having acute coronary syndrome (ACS). She was transported immediately to the catheterization laboratory. Coronary angiography, performed via the right femoral artery, showed subtotal occlusion and thrombus-like filling defect in the mid portion of the RCA (Figure 1). The left circumflex (LCx) and the left anterior descending artery (LAD) exhibited only mild irregularities without significant stenosis (Figure 2). Percutaneous coronary intervention was not performed. The patient was transferred to the coronary care unit where continuous infusion of tirofiban (0.4 mcg/kg/min bolus) over 30 minutes followed by 0.25 mcg/kg/min for 24 hours, heparin (1000 U/hours), and the oral combination of clopidogrel (75 mg/d before 300 mg bolus), plus aspirin (100 mg/day) was re-instituted. We kept the activated clotting time between 250 and 300 s during the infusion of heparin and tirofiban. A control coronary angiogram obtained three days later showed total dissolution of the coronary thrombus and normal clearance of the culprit vessel and TIMI-III flow was seen (Figure 3). Also, there was no distal embolization. Laboratory analysis showed leukocytosis (WBC: 17300/mm^3^) and thrombocytosis (platelet count:1.243.000/mm^3^). Bone marrow aspirate revealed myeloid and megakaryocytic hyperplasia with mild degree fibrosis (Figure 4). Bone marrow biopsy of the patient was consistent with typical myeloproliferative disease and aspiration samples were sent to the Molecular Biology Laboratories of the Medical Biology Department, Ege University. Genomic DNA was extracted from peripheral blood leukocytes by using the High Pure PCR Template Preparation Kit (Roche Applied Science, Mannheim, Germany) and stored at -20°C until use. Gene polymorphism and mutation analysis was either carried out by commercial available kits (LightCycler Factor V Leiden Mutation Detection Kit, and LightCycler Prothrombin (G20210A) Mutation Detection Kit, Roche Applied Science, Mannheim, Germany; LightMix Kit JAK2V617F genomic, LightMix for the detection of human MTHFR C677T, and LightMix for the detection of human Plasminogen Activator Inhibitor 4G/5G, TIB MOLBIOL, berlin, Germany) or in the case of the Factor XII C46T gene polymorphism by a protocol published from Tirodo, I. et al. [6]. All experiments were carried out on the LightCycler™ Instrument ver.2.0 (Roche Applied Science; Mannheim, Germany).

Total RNA was isolated from bone marrow cells of the subject by using the High Pure RNA Isolation Kit (Roche Applied Science, Mannheim, Germany) and stored at -86°C until use. Quantitative bcr-abl analysis was performed with the LightCycler t(9;22) Quantification Kit on the LightCycler™ Instrument ver.2.0 (Roche Applied Science; Mannheim, Germany).

Bone marrow specimens were negative for the presence of the bcr/abl transcript. JAK2V617F mutation, Factor V Leiden (G 1691 A), Prothrombin gene (G 20210 A) mutations, MTHFR (C 677 T), Factor XIII (C 46 T), and PAI-1 (4G/5G) genotype polymorphism analysis were performed to determine the potential hereditary risk factors for thrombophilia. PAI-1 4G/4G homozygote polymorphism and JAK2V617F heterozygote mutation were detected. Bone marrow findings, clinical presentation and laboratory analysis confirmed the diagnosis of essential thrombocythemia. Hydroxyurea was given to control the leukocyte and thrombocyte count.

## Discussion

Intracoronary thrombus (ICT) may occur in ACS spontaneously, sometimes as a complication of interventional procedures and frequently superimposes on an existing atherosclerotic lesion [7]. However, an ICT or myocardial infarction with normal coronary arteries is a syndrome resulting from numerous conditions, but the exact cause in a majority of patients remains unknown. Possible mechanisms include embolism, vasospasm, nonatherosclerotic coronary diseases, hypercoagulable states, trauma, an imbalance between oxygen demand and supply, intense sympathetic stimulation, and endothelial dysfunction [8]. Because of RCA was determined as normal on recent angiogramme, the possible mechanism of thrombus in our case is considered due to hypercoagulablity that induced essential thrombocythemia.

Myeloproliferative disorders are a heterogeneous group of diseases characterized by excessive proliferation of cells originated from the myeloid lineage. Chronic myeloid leukemia, polycythemia vera, essential thrombocythemia and myelofibrosis are leading forms of the disorder. ET is a clonal disorder of a multipotent stem cell resulting in thrombocytosis, leukocytosis [9,10]. Patients with ET are thought to be at increased risk of thromboembolic events. Arterial ischemic complications may occur in these patients [10]. Elevated platelet count is regarded as risk factor, although the significance of these parameters has not been confirmed by clinical studies [11,12].

At diagnosis, 5% of ET patients show chromosomal abnormalities [13]. The most common cytogenetic abnormalities detected by conventional cytogenetic techniques are deletions in the long arms of chromosomes 20 and 13, duplication of 1q, and trisomies of chromosomes 8 and 9 [13]. Kralovics et al. [13] found loss of heterozygosity to be present in chromosome 9p, where JAK2 resides. JAK2 is a member of the Janus family of cytoplasmic non-receptor tyrosine kinases. A guanine-to-thymidine substitution, which results in a substitution of valine for phenylalanine at codon 617 of JAK2 (JAK2V617F), is responsible for the constitutively activity of this tyrosine kinase that activates signal transducer and activator of transcription (STAT), mitogen activated protein kinase (MAPK) and phosphotidylinositol 3-kinase (PI3K) signalling pathways, and transforms haematopoietic progenitor cells [14-16]. The discovery of JAK2V617F has led to the development of selective JAK2 inhibitors for the treatment of PV, ET and PMF (6, 16). Its exon 14 harbors JAK2V617F mutation, recently reported to be present in approximately 50% of ET. In addition, JAK2 exon 12 mutation in PV2, and a thrombopoietin receptor MPLW515L/K mutation in ET and myelofibrosis [17] are novel mutations that have been described in JAK2V617F mutation negative patients. In ET in particular, approximately 50% of the patients are JAK2 mutation negative and only a small fraction (less than 5%) has MPL mutation. However, recent studies have showed that no significant differences for clinical complications except megakaryocyte counts between the patients with JAK2 V617F-positive and -negative [18].

Sarecka et al. reported the relationship between 5G allele of PAI-1 gene and the coronary artery disease. This data and the previously recorded data and Hindorff et al. reported that 4G allele was associated with a reduced risk of myocardial infarction and in the premenopausal women group 5G allele was correlated with a higher risk of infarct [19-21]. Böttiger et al. found no association between the 4G/5G promoter polymorphism of the PAI-1 gene and angiographic restenosis or clinical outcome after coronary artery stenting in a large consecutive cohort of patients [22]. Our case had homozigous PAI-1 4G/4G and heterozygote JAK2V617F polymorphisms.

Douste-Blazy and associates reported that in cases of ET, pathophysiologic mechanisms of thrombus formation in different coronary arteries were 1) activation of thrombocytes as a result of endothelial damage, 2) extended arterial spasms and as a result formation of thrombus, 3) increased procoagulant activity of thrombocytes, 4) changes in glycoproteins on the surface of thrombocytes, and 5) possible deficiency of selective lipooxygenase [23]. ACS, developing in cases of ET, was not a result of atherosclerotic plaque rupture and was characterized by formation of the primary thrombus due to increased numbers of platelets, frequently in intramural arteries and rarely in epicardial arteries [24]. In these patients, coronary angiograms are frequently found to be normal [25]. No specific treatment of ACS, in patients with ET is reported. In the literature, only rare cases of application of conventional treatment or coronary angioplasty are found [26]. In this case, tirofiban, a GP IIb/IIIa receptor blocker, was preferably used, taking into account the pathophysiology of the disease.

Therefore, it may be thought that, in patients with ET, thrombolytic resistance treatment could develop in ACS generated by primary platelet rich thrombus, and blocking the GP IIb/IIIa receptors could break the continuing chain of aggregation and activation. Clinical improvement following tirofiban treatment, noticed without any complication was observed. Coronary angiograms support our concept of the use of GP IIb/IIIa receptors blockers in this case.

Identification and successful treatment of similar cases with tirofiban will help in further understanding of the pathophysiology of acute coronary syndromes in patients with ET.

## Competing interests

The authors declare that they have no competing interests.

## Consent

Written informed consent was obtained from the patient for publication of this case report and accompanying images. A copy of the written consent is available for review by the Editor-in-Chief of this journal.

## Authors' contributions

EA carried out management of patient in coronary care unit and participated in second coronary angiography. NT collected material about similar cases. FS carried out the hematological tests. BK carried out the genetic examination. SS collected material about similar cases. IT performed first coronary angiography. UT performed second coronary angiography. NO participated in pathological examination of specimens obtained from bone marrow. EE participated in the sequence alignment. All authors read and approved the final manuscript.
